# Exploring the Potential Role of Rosmarinic Acid in Neuronal Differentiation of Human Amnion Epithelial Cells by Microarray Gene Expression Profiling

**DOI:** 10.3389/fnins.2019.00779

**Published:** 2019-07-24

**Authors:** Farhana Ferdousi, Kazunori Sasaki, Yoshiaki Uchida, Nobuhiro Ohkohchi, Yun-Wen Zheng, Hiroko Isoda

**Affiliations:** ^1^Alliance for Research on the Mediterranean and North Africa, University of Tsukuba, Tsukuba, Japan; ^2^National Institute of Advanced Industrial Science and Technology, Tsukuba, Japan; ^3^School of Integrative and Global Majors, University of Tsukuba, Tsukuba, Japan; ^4^Department of Gastrointestinal and Hepato-Biliary-Pancreatic Surgery, Faculty of Medicine, University of Tsukuba, Tsukuba, Japan; ^5^Faculty of Life and Environmental Sciences, University of Tsukuba, Tsukuba, Japan

**Keywords:** human amnion epithelial cell, rosmarinic acid, microarray analysis, system biology, neural induction, neuronal differentiation, natural compound

## Abstract

In the present study, we conducted microarray gene expression profiling to explore the direction of differentiation of human amnion epithelial cells (hAECs) treated with rosmarinic acid (RA). hAECs have several clinical advantages over other types of stem cells, including availability, low immunogenicity, low rejection rate, non-tumorigenicity, and less ethical constraint. On the other hand, RA is a phenolic compound having several health benefits, including neuroprotective and antidepressant-like activities. In this study, hAECs were isolated from discarded term placenta and were treated with 20 μM RA for 7 days. Microarray gene expression profiling was conducted for three biological replicates of RA-treated and untreated control cells on day 0 and day 7. Gene set enrichment analysis, and gene annotation and pathway analysis were conducted using online data mining tools GSEA and DAVID. Gene expression profiling showed that RA treatment biased hAECs toward ectodermal lineage progression, regulated transcription factors involved in neuronal differentiation, regulated neural specific epigenetic modifiers and several extracellular signaling pathways of neural induction, and significantly inhibited Notch signaling pathway. Gene expression profiling of RA-treated hAECs reveals for the first time a potential role of RA in neural induction and neuronal differentiation of hAECs. Having a naturally occurring compound as differentiation inducer as well as a readily available source of stem cells would have great advantages for the cell-based therapies. Findings from our genome-wide analysis could provide a foundation for further in-depth investigation.

## Introduction

Human amnion epithelial cell is gaining interest as a novel alternative source of stem cells in the field of regenerative cellular therapy. hAECs express surface markers of both ESCs and MSCs and have similar functional properties of both of the stem cells. Additionally, hAECs have several advantages over other types of stem cells. Because of their embryonic origin, hAECs retain their pluripotent potential and can be differentiated into all three germ layers under proper condition. However, unlike other stem cells, they do not form teratomas *in vivo*. Most importantly, hAECs have low immunogenicity and a wide range of immunomodulatory properties. These cells can be obtained from discarded term placenta and are, therefore, readily available and devoid of any ethical constraint. These unique properties of hAECS offer significant practical advantages for potential clinical applications (Miki et al., 2005; Miki and Strom, 2006; De Coppi et al., 2007). In several studies, hAECS were induced to differentiate into specific cell types, including hepatocytes, adipocytes, myocytes, cardiomyocytes, osteocytes, pancreatic cells, as well as neural and glial cells. However, it was reported that hAECs are primarily committed to ectodermal lineage, and can express several neural markers even before the induction of differentiation (Pan et al., 2006). Treatment with retinoic acid and basic fibroblast growth factor was also reported to induce neural markers expression in hAECs (Niknejad et al., 2010). Sakuragawa et al. (1996) reported that under certain conditions, differentiated hAESCs can synthesize and release acetylcholine, catecholamines, dopamine, neurotrophic factors, and noggin. Use of hAECs in cellular therapies of spinal cord injuries has been reported promising because of their ability to induce neuronal differentiation and neurotransmitter secretion. Thus, hAECs have new therapeutic potential for several neurodegenerative disorders that affect the central nervous system (McDonald et al., 2011; Miki, 2011).

Currently, different synthetic and semi-biological cytokines, recombinant growth factors, and proteins are used for stem cell proliferation and differentiation. Most of these substances have several drawbacks. They may show toxic effects, have a possible risk of rejection, and can cause malignancy (Marion and Mao, 2006; Raghavan et al., 2015). These reagents are rapidly degradable and are required to replace continuously, making the whole procedure highly expensive, hence limiting their use in therapeutic tissue engineering. Therefore, a new research stream has been evolving to use natural medicinal plant extracts as stimulants of stem cells because of their high availability, low toxicity, and minimum side effects (Raghavan et al., 2015). Plant-derived extracts are rich in polyphenols, and flavonoids, and have proven health beneficial effects. A few studies have reported the differentiation potential of some herbal extracts on different types of stem cells (Udalamaththa et al., 2016). Additionally, several plant extracts and their active components showed potential effects in neuronal differentiation and proliferation, like phytochemicals from *Butea superba* in stem cells (Arai et al., 2013), flavonoid component from *Scutellaria baicalensis* in C17.2 cells (Li et al., 2012), acetic acid extract of *Mucuna gigantea* in bone marrow-derived hMSCs (Kongros et al., 2012), *Salvia miltiorrhiza* extract on Wharton jelly derived hMSCs (Ma et al., 2005). However, to our knowledge, there is no study to date reporting the effect of any natural compounds on hAECs differentiation.

As a part of our continued research efforts to explore beneficial effects of naturally occurring compounds of plant origin from the Mediterranean region, we have been investigating the effects of different natural compounds on the direction of hAECs differentiation. Rosmarinic acid is a phenolic compound and an ester of caffeic acid. It is commonly found in several culinary herb species, like *Rosmarinus officinalis* (rosemary), *Ocimum basilicum* (basil), *Ocimum tenuiflorum* (holy basil), and *Melissa officinalis* (lemon balm). It has several health benefits, like anti-inflammatory, antioxidative, and neuroprotective effects (Alagawany et al., 2017). We have previously reported that ethanolic extract of *R. officinalis*, as well as RA itself, has neuroprotective and anti-depressant like activities both *in vitro* and *in vivo* (Sasaki et al., 2013; Kondo et al., 2015). We also reported neuronal cell differentiation induction effects of essential oil of *R. officinalis* (Villareal et al., 2017).

Microarray gene expression profiling is a useful tool to explore genome-wide expression patterns that are activated during studied biological conditions and provides a foundation for further examination of molecular mechanism and regulatory pathways. In this study, we treated three dimensional (3D) spheroids of hAEC with 20 μM of RA for 7 days and conducted microarray analysis to explore its effects on the direction of hAECs differentiation.

## Materials and Methods

### Extraction of Amnion Epithelial Cells

Amnion epithelial cells were isolated from delivered term placenta. The amnion was aseptically separated from the chorion and was washed with 200 mL of Hank’s Basic Salt Solution – Calcium and Magnesium Free (CMF-HBSS) to remove blot clots on the membrane. The amnion was then cut into smaller pieces using surgical scissors and placed into 50 mL conical tubes. A pre-digestion buffer (CMF-HBSS, 0.5 mM EGTA, 20 mL) was added to the tubes. The amnion was gently rocked in the solution for 30 s and was transferred to new 50 mL conical tubes. Twenty mL of pre-digestion buffer was added to the amnion tissue and was incubated at 37°C for 10 min. The pre-digestion buffer was discarded and 20 mL of 0.05% trypsin-EDTA was added to the tissue. The tubes were incubated for 40 min at 37°C and then placed on ice after incubation. Two volumes of Dulbecco’s Modified Eagle Medium (DMEM, 10% FBS, 1% Penn-Strep) was added to the trypsin digest and centrifuged at 200 g for 10 min at 4°C. The supernatant was discarded and the pellet was resuspended in 10 mL of DMEM. Small tissue aggregates were removed by filtration through a 100 μm filter. The cell suspension was collected and the membrane was discarded.

### Cell Culture Maintenance

Amnion epithelial cells were maintained in Placenta Epithelial Cell Basal Medium (PromoCell, Cat. #C-26140). Cells were constantly monitored with media change every 2–4 days. To subculture AECs, the plates were first washed twice with 10 mL of PBS. The PBS was discarded and 3 mL of pre-digestion buffer (pre-warmed to 37°C) was added to the plate. The plate was incubated at 37°C for 5 min. Five mL of 0.05% (w/v) trypsin-EDTA (pre-warmed to 37°C) was then added to the plate and incubated at 37°C for 10 min. Five mL of DMEM was added to stop the reaction. The cell suspension was placed into conical tubes and centrifuged at 200 RPM for 4 min at 4°C. The supernatant was discarded and the pellet was resuspended in DMEM. The cell suspension was again centrifuged at 200 RPM for 4 min at 4°C. The supernatant was discarded and the cells were resuspended in the placental cell medium. The cells were then seeded onto new cell culture dishes.

### Preparation of 3D Culture Plates

Lipidure TM (NOF Corporation, Cat. #CMS206; 400 μL) solution (50 mg in 10 mL absolute ethanol) was placed into each well of the 3D culture plate (Elplasia^TM^, Kuraray Co., Ltd., Cat #RB 500 400 NA 24) and was allowed to sit for 2 min. The Lipidure TM solution was aspirated out and the plate was dried for 3 h. After drying, 400 μL of PBS was placed in each well and the plate was centrifuged at 2000 g for 15 min at room temperature. The plate was observed under the microscope to ensure that there were no bubbles in the wells. The PBS was then discarded and the wells were washed twice with 400 μL of PBS. The plates were then stored in the cell culture incubator until use.

### Spheroid Formation and Compound Supplement

From each well of the 3D culture plate, 300 μL of PBS was aspirated. Spheroids were formed by seeding 1 × 10^6^ AECs in Placenta Basal Epithelial Cell Medium into each well of the 24-well plate. After the initial 24 h culture, the medium was changed with 20 μM of RA (FUJIFILM Wako Pure Chemical Corporation, Japan) every 48 h for three times for the treatment samples. Day 0 control (D0) samples were collected before adding the treatment to the cells. Control samples were maintained in the Placental medium that was also changed every 48 h. Finally, we collected the treatment (T7) and control (D7) samples from 1-week culture.

### RNA Extraction and Quantification

For RNA extraction, Isogen (Nippon Gene Co., Ltd., Toyama, Japan) was added and the cell suspensions were centrifuged for 5 min at 500 g and the pellet was stored at −80°C until use. The integrity of RNA was quantified using NanoDrop 2000 spectrophotometer (Thermo Fisher Scientific, Wilmington, DE, United States).

### Microarray Expression and Data Analysis

We prepared RNA samples for gene expression profiling analysis with GeneChip^®^ 3′ Expression Arrays using 3′ IVT PLUS Reagent Kit (Affymetrix Inc., Santa Clara, CA, United States). Two hundred and fifty ng of total RNA from each sample was used to generate amplified and biotinylated cRNA from poly (A) RNA in a total RNA sample according to the user manual. IVT Incubation time was 16 h. GeneAtlas^®^ Hybridization, Wash and Stain Kit was used for hybridizing 3′ IVT Array Strips according to the user manual (P/N 08-0306). Human genome array strips (HG-U219) were hybridized for 16 h in a 45°C incubator, washed and stained and finally imaging was done with the GeneAtlas Fluidics and Imaging Station. HG 219 array strip can determine the expression profile of 49386 genes. Microarray expression profiling was conducted for two biological replicates between T7 and D0 samples and three biological replicates between T7 and D7 samples.

The raw data were normalized using Expression Console Software provided by the Affymetrix following robust multichip average (RMA) algorithm^1^. Subsequent analysis of the gene expression data was carried out in the freely available Transcriptome Analysis Console (TAC) version 4 (Thermo Fisher Scientific Inc.). Genes with a fold change > 1.1 (in linear space) and *p*-Value < 0.05 (one-way between-subject) were considered as DEGs. Further analysis was conducted using an online data mining tool DAVID (Database for Annotation, Visualization, and Integrated Discovery, version 6.8). We used “Functional Annotation” tool of DAVID to identify the most relevant biological terms, including GO terms, biological pathways, tissue expression, and disease associations (Huang da et al., 2009). We also performed gene set enrichment analysis (GSEA) for the top 3000 DEGs between T7 and D0 hAECs and for all DEGs between T7 and D7 hAECs to test whether *a priori*-defined groups of genes associated with neural differentiation were significantly related to the DEGs between RA-treated and control cells on day 0 and day 7^2^ (Subramanian et al., 2005).

### Ethics Approval

The protocol was reviewed and approved by the Ethical Review Committee of the University of Tsukuba. Informed written consent was obtained from the mothers who donated the placenta after delivery.

## Results

We treated 3D hAECs with 20 μM of RA for 7 days and compared the gene expression pattern between untreated control cells on day 0 and day 7 and RA-treated cells. We identified DEGs, respectively, in two (T7 vs. D0) and three (T7 vs. D7) biological replicates and explored the biological events that took place during seven days of RA treatment.

### Characteristics of AECs and DEGs

Several stem cell markers were expressed highly in the undifferentiated hAECs, like SSEA-4 (99.2%), Tra-1-60 (71.1%), Tra-1-81 (13.7%), EPCAM (75.4%), E-cadherin (99.9%), and Lectin (98.1%) (Supplementary Figure 1). Additionally, AEC spheroids (3D culture) highly expressed the stemness-related genes compared to the 2D counterpart. On the 7th day of culture, 3D spheroids expressed *OCT-4*, *NANOG*, *LIN28*, *SOX-2*, *C-MYC*, and *KLF4* significantly higher than 2D cells (Supplementary Figure 2).

**FIGURE 1:**
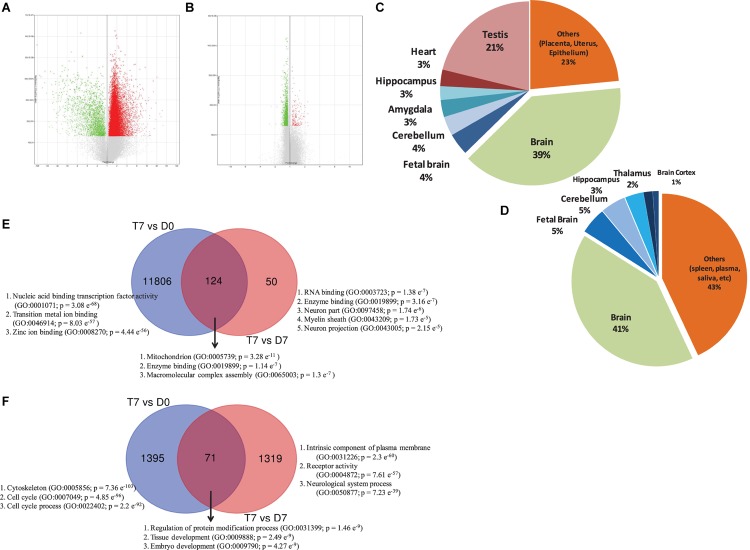
Volcano plot displaying DEGs between **(A)** RA-treated (T7) and day 0 untreated-control (D0) hAECs, and **(B)** RA-treated (T7) and day 7 untreated-control (D7) hAECs. The vertical axis (*y*-axis) corresponds to -log10 *p*-Value of the ANOVA *p*-Values, and the horizontal axis (*x*-axis) displays linear fold change. The red dots represent the up-regulated genes; the green dots represent the downregulated genes. Pie chart showing the enriched (*p* < 0.05) tissue expressions by the DEGs between **(C)** RA-treated (T7) and day 0 untreated-control (D0) hAECs, and **(D)** RA-treated (T7) and day 7 untreated-control (D7) hAECs. Venn diagram showing common and unique sets of DEGs between each exposure. Blue circles denote DEGs between RA-treated (T7) and day 0 untreated-control (D0), red circles denote DEGs between RA-treated (T7) and day 7 untreated-control (D7). **(E)** Venn diagram for upregulated DEGs. **(F)** Venn diagram for downregulated DEGs. Significantly enriched gene ontology (GO) have been listed for each set of DEGs.

Although a huge number of genes were differentially expressed between treatment and control cells on day 0 (T7 vs. D0), there was not much change in gene expression between treatment and control cells on day 7 (T7 vs. D7), as we did not add any additional supplements to induce differentiation (Supplementary Figure 3). To understand the integrative response of hAECs, including how they actively adjust transcriptome after RA treatment through GO annotation groups, we assigned the cutoff for DEGs to be >1.1 (fold change); *p*-Value < 0.05. We found 23 976 genes between T7 and D0 samples and 1740 genes between T7 and D7 samples were consistently differentially expressed. After deleting duplicates and unnamed genes, we identified 13 396 DEGs between T7 and D0 samples, among which 11 930 genes were upregulated and 1466 were downregulated. On the other hand, there were 1572 DEGs between T7 and D7 samples, among which 176 genes were upregulated and 1396 genes were downregulated. Volcano plots show the distribution of fold change and significance levels of the DEGs (Figures 1A,B). We found that nearly 60% of DEGs were brain-specific (Figures 1C,D). We assumed RA treatment could induce, at least in part, neuronal differentiation.

### Significantly Enriched Gene Sets and GO

**FIGURE 2:**
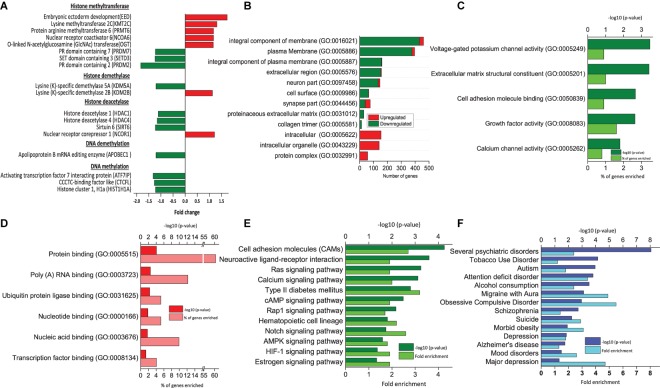
Functional analysis of DEGs between RA-treated (T7) and day 7 untreated control (D7) hAECs. **(A)** Changes in the expression of epigenetic modifiers by RA treatment (T7) compared with untreated-control on day 7 (D7); **(B)** Significantly enriched cellular components (*p* < 0.05; modified Fisher’s exact test); **(C)** Top molecular functions as per *p*-Value (modified Fisher’s exact) by downregulated genes. **(D)** Top molecular functions as per *p*-Value (modified Fisher’s exact) by upregulated genes. **(E)** Significantly enriched KEGG pathways by downregulated genes (*p* < 0.05; modified Fisher’s exact test). **(F)** Significantly enriched psychological and neurological diseases (*p* < 0.05; modified Fisher’s exact test).

Gene set enrichment analysis showed that several priori-defined hallmark gene sets associated with cell cycle, such as G2/M checkpoint and E2F targets, along with GO of cell differentiation (GO: 0045595) and neurogenesis (GO: 0022008) were significantly enriched by the DEGs between T7 and D0 cells (Table 1). When compared between T7 and D7 cells, significantly enriched GO were cell differentiation (GO: 0045595), neurogenesis (GO: 0022008), and neuron differentiation (GO: 0030182). Top gene sets by the unique upregulated DEGs between T7 and D0 were nucleic acid binding (GO: 0001071), metal ion binding (GO: 0046914), and Zinc ion binding (GO: 0008270); while top significantly enriched gene sets by the unique upregulated DEGs between T7 and D7 were RNA binding (GO: 0003723), enzyme binding (GO: 0019899), and neuron part (GO: 0097458; Figure 1E). Figure 1F shows the top significantly enriched gene sets by the unique sets of downregulated DEGs between each exposure. Detailed functional analysis of DEGs between T7 and D0 and between T7 and D7 is presented in Figure 2 and Supplementary Figure 4, respectively. Supplementary Table 1 listed the fold changes and *p*-Values of important DEGs. Top ten upregulated DEGs between T7 and D0 are significantly associated with tissue development (GO: 0009888, *p* = 2.41 e^–7^) and epithelial cell differentiation (GO: 0030855, *p* = 2.65 e^–6^).

**TABLE 1:** Significantly enriched gene sets by DEGs between RA treated and control cells.

Gene Set Name^*^	**Systematic name, exact source**	**No. of genes in set**	**No. of genes in overlap**	***p*-value**	**Adjusted *p*-value**
**Significantly enriched gene sets by DEGs between RA treated (T7) and control cells (D0)**					
HALLMARK^1^_G2M_CHECKPOINT^2^		200	81	2.56 e^–46^	1.28 e^–44^
HALLMARK_E2F_TARGETS^3^		200	70	2.38 e^–35^	5.94 e^–34^
HALLMARK_EPITHELIAL_MESENCHYMAL_TRANSITION		200	58	1.01 e^–24^	1.01 e^–23^
GO_CELL_CYCLE	M14460, GO:0007049	1316	284	5.72 e^–84^	3.39 e^–80^
GO_CYTOSKELETON	M17350, GO:0005856	1967	351	2.33 e^–80^	6.95 e^–77^
GO_REGULATION_OF_CELL_DIFFERENTIATION	M13423, GO:0045595	1492	236	3.55 e^–44^	5.88 e^–42^
GO_NEUROGENESIS	M13908, GO:0022008	1402	206	6.57 e^–34^	5.05 e^–32^
**Significantly enriched gene sets by DEGs between RA treated (T7) and control cells (D7)**					
GO_NEUROLOGICAL_SYSTEM_PROCESS	M10152, GO:0050877	1242	133	3.36 e^–35^	1.71 e^–32^
GO_NEUROGENESIS	M13908, GO:0022008	1402	141	2.47 e^–34^	1.09 e^–31^
GO_REGULATION_OF_CELL_DIFFERENTIATION	M13423, GO:0045595	1492	142	5.11 e^–32^	1.8 e^–29^
GO_NEURON_DIFFERENTIATION	M15330, GO:0030182	874	92	4.8 e^–24^	6.81 e^–22^

^*^GSEA online software (http://software.broadinstitute.org/gsea/index.jsp). ^1^Hallmark gene sets summarize and represent specific well-defined biological states or processes and display coherent expression. ^2^Genes involved in the G2/M checkpoint, as in progression through the cell division cycle. ^3^Genes encoding cell cycle related targets of E2F transcription factors.

### RA Treatment Biased hAECs Toward Ectodermal Lineage Progression

In the RA-treated cells, there was no expression of stem cell marker genes, indicating that the cells lost their stemness and proceeded toward differentiation. Several endoderm marker genes, like *GATA1*, *HNF4A*, *NKX3*, and *SOX17* were significantly upregulated when compared between T7 vs. D0 cells, whereas significantly downregulated when compared between T7 and D7 (Supplementary Table 1). Similarly, some mesoderm marker genes, namely *MEST*, *MEOX1*, *MESDC1*, *DLL3*, *MESP1*, and *FOXF1* were also downregulated when compared between T7 and D7. The expression of some known ectoderm marker genes, including *OTX*, *SOX1*, and *PAX6*, were significantly upregulated when compared between T7 and D0 hAECs, but no change in expression was observed when compared between T7 and D7 hAECs. However, a less well-known early ectoderm development marker gene *EED* was expressed significantly higher in both exposures (1.64 and 1.73 fold in T7 vs. D0 and T7 vs. D7, respectively). Eed is a polycomb protein and a core protein of PRC 2. It is expressed by neural stem cells (NSCs) and is necessary for subventricular zone NSC activation, and neurogenesis (Sun et al., 2018). When compared between T7 and D7 hAECs, GSEA showed that 91 DEGs matched the set “PRC2 targets” (*p*-Value = 6.47 e^–33^), and 119 DEGs matched the set “Eed targets” (*p*-Value = 2.17 e^–33^) identified in human ESCs (Ben-Porath et al., 2008). There was upregulation of *BCOR* (1.28 fold), a transcriptional corepressor of *BCL6* (Huynh et al., 2000). Bcl6 controls neurogenesis through epigenetic repression of selective Notch targets. There was downregulation of *DPPA4* (1.32 fold), a possible inhibitor of embryonic cell differentiation into primitive ectoderm lineage.

### RA Treatment Regulated TFs Involved in Neuronal Differentiation

Analysis of up- and downregulated TFs between T7 vs. D7 hAECs revealed TFs involved in neuronal differentiation were highly regulated (*TCF12*, *HNRNPD*, *NLK*, and *SMAD5*). Tcf12 (also known as *HEB* in human) is a member of bHLH E-proteins that are heterodimeric partners of neural-specific bHLH proteins (Uittenbogaard and Chiaramello, 2002; Mesman and Smidt, 2017). Tcf12 plays an important role both in the early cell-fate commitment of neural progenitors and in subset specification. On the other hand, *HELT*, a bHLH transcriptional repressor of neuronal differentiation, was downregulated (Nakatani et al., 2004). Nlk is a positive effector of the non-canonical Wnt pathway and enhances neural fate by limiting epidermal choice. Additionally, Smad5 activity is reported to be important for the maintenance of neural progenitor cells. Loss of Smad 5 along with Smad 1 could reduce the number of newly generated neurons and forced cell cycle exit and premature neurogenesis of neural progenitors. On the other hand, *ASCL1* that plays a role at early stages of development of specific neural lineages probably through DLL4-NOTCH signaling activation was downregulated.

Several TFs involved in epidermal differentiation (*TCF7L1*), epithelial cell growth (*FOXE3* and *EHF*), lymphocyte development (*ZBTB17*), lymphoid cell fate determination (*MAML1*), erythroid development (*GATA1*), liver development (*HNF4A*), hair follicle differentiation (*HOXC13*), kidney cell differentiation (*PAX2*), limb development (*SP8*), hematopoietic stem cell differentiation (*EGR1*) (Min et al., 2008), and cardiovascular development (*HEYL*), were downregulated.

### RA Treatment Regulated Neural Specific Epigenetic Modifiers

Modification of neural development genes at the DNA level plays an important role in neural induction (Yao and Jin, 2014). We analyzed the effect of RA treatment on the expression pattern of genes that are involved in epigenetic regulation during neural induction (T7 vs. D7). We found that several genes of histone methyltransferases were both up- and downregulated (Figure 2A). Other than *EED*, a lysine-specific methyltransferase (*PRMT6*), and an arginine-specific methyltransferase (*KMT2C*) was upregulated. On the other hand, lysine-dependent three other methyltransferases, namely *PRDM2* and *7* and *SETD3* were downregulated. Both activating and repressing of histone methylation marks is necessary for the rapid activation of genes during differentiation. Similarly, we found lysine (K)-specific histone demethylase *KDM2B* was upregulated, whereas *KDM5A* was downregulated. Most interestingly, we found genes of HDAC complex were downregulated (*HDAC1*, *HDAC4*, and *SIRT6*). It was reported that transcriptional repression mediated by EED involves histone deacetylation (van der Vlag and Otte, 1999). HDACs are involved in the transition from deep layer neuron to upper layer neuron and oligodendrocyte differentiation. However, there was no change in the gene expression of DNA methyltransferases. Usually, DNA methylation takes place from mid-stage to late stage of neuronal differentiation. We assume that as we collected the sample on the 7th day of culture, it was too early to detect any DNA methylation activity directly (Namihira et al., 2008; Murao et al., 2016).

### RA Treatment Regulated Neural Specific Epithelial-Mesenchymal Transition

Epithelial-Mesenchymal Transition is a common phenomenon during embryonic development, however, is less explained during neural induction. One of the significantly enriched hallmark gene sets by the DEGs between T7 and D0 is EMT (number of DEGs = 58, *p* = 1.01 e^–24^, Table 1). The previous study reported that formation of neuroectoderm from hESCs involves genes of extracellular matrix components (Wang et al., 2012). GO analysis of DEGs between T7 and D7 revealed that significantly enriched cellular components by downregulated genes are an integral component of the plasma membrane, extracellular region, cell surface, and collagen trimer. Significantly enriched cellular components by upregulated genes are intracellular organelle and protein complex (Figure 2B). A similar pattern of gene expression is observed during the early stage of neuronal differentiation. We found downregulation of genes from *CADHERIN* family (*CDH6*, *CDH16*, and *CDHR5*), *COLLAGEN* family (*COL11A1*, *COL13A1*, *COL14A1*, *COL4A1*, *COL4A2*, and *COL9A1*). Moreover, genes associated with tight junction (*CLDN18* and *EPB41L5*), desmosome (*KAZN*), cell surface receptor (*CD74*, *CSPG4*, and *DCSTAMP*), cell polarity (*ARHGAP24*), and vesicle transport (*FYCO1* and *SPIRE 1*) were consistently downregulated (Supplementary Table 1), indicating potential role of RA treatment in EMT in neural induction (Pankratz et al., 2007; Park et al., 2012).

### RA Treatment Regulated Several Extracellular Signaling Pathways of Neural Induction

Several extracellular signaling pathways are well established in a number of proposed models of neural induction. We examined the expression profile of genes related to WNT, TGF-β, and BMP signaling pathways (Doe, 2008). When compared between T7 and D7 hAECs, we found that several genes of the canonical WNT pathway (WNT/β catenin) were downregulated, such as *BTRC*, *CCNY*, *FZD5*, *RSPO1*, *RSPO4*, *SIX3*, *WISP3*, and *WNT5B*. On the other hand, a positive effector of the non-canonical WNT (WNT/ calcium) signaling pathway *NLK* was upregulated. BMP/TGF-β signaling pathway related genes, like *ACVRL1*, *ASPN*, *CHRD*, and *SLC39A5* were downregulated. *BMP8A*, *BMP8B*, *BMPER*, and *TGF*-β*2* were also downregulated. On the other hand, the major intracellular BMP signaling pathway component *SMAD 5* was upregulated (Leung et al., 2013; Huang et al., 2016).

### RA Treatment Significantly Inhibited Notch Signaling Pathway

Notch is considered as a molecular switch in NSCs (Zhou et al., 2010; Imayoshi and Kageyama, 2011). Loss of Notch signaling in NSC leads to increased cell cycle exit and decreased progenitor pool. When compared between T7 and D0 hAECs, several genes associated with cell cycle and notch signaling were significantly upregulated (Table 1 and Supplementary Table 1). However, when compared with T7 and D7 hAECs, we found that RA treatment could significantly downregulate several genes related to the Notch pathway indicating that RA treatment could lead to cell cycle exit and decreased progenitor pool. Notch receptor (*NOTCH2*), transmembrane ligand of Notch receptors (*DLL1* and *DLL3*), a positive regulator of Notch (*DTX1*), a downstream effector of Notch signaling (*HEYL*), transcriptional coactivator for NOTCH (*MAML1* and *SBNO2*), a downstream target of Notch signaling pathway (*PKMYT1*) were downregulated.

Furthermore, RA might also work on hAECs through downregulating neuroactive ligand-receptor interaction pathways, Ras signaling pathway, the cAMP signaling pathway, and AMPK signaling pathway (Figure 2E). Ras signaling pathway is an upstream pathway for MAPK, Rap 1 pathway. Similarly, cAMP is upstream of the Hedgehog pathway. All of these pathways are known to be regulated at different stages of neuronal differentiation and are balancing between self-renewal and differentiation (Doe, 2008).

### RA Treatment Significantly Enriched Several Neurological and Psychological Diseases

We used the functional annotation tool of DAVID for annotating genome-wide data with disease associations using the GAD (Osborne et al., 2009). Several neurological and psychological diseases were significantly enriched, particularly by the downregulated genes, such as Schizophrenia, obsessive-compulsive disorder, migraine with aura, alcohol consumption, bulimia, attention deficit disorder, autism, and tobacco use disorder (Figure 2F). Moreover, major depression, mood disorders, depression, and suicidal tendency related genes were also regulated.

## Discussion

**FIGURE 3:**
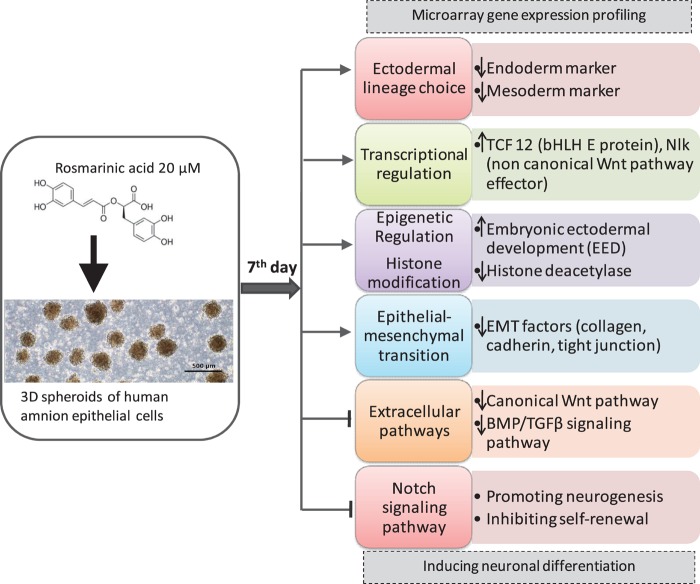
Summary of the Effect of RA in hAECs differentiation.

Placenta-derived hAECs is gaining interest in the field of regenerative medicine to overcome the current challenges of existing cell-based therapies, such as cell resources, invasive extraction procedures, immunological rejection, tumorigenesis and ethical challenges (Miki, 2011). Previous studies reported that cultured human amnion cells were able to express markers characteristic of NSCs (nestin, Musashi-1, and polysialylated neural adhesion molecule), neural cells (TH, AchT, NeuN, MAP-2, and myelin basic protein), astrocytes (GFAP), and oligodendrocytes under optimal differentiation condition (Pan et al., 2006). However, there is no study on the effect of natural compounds on hAECs differentiation. In the present study, we aimed to evaluate microarray gene expression profiling of RA-treated 3D spheroids of hAECs to explore its neuronal differentiation-inducing effect and to provide a foundation for further examination of molecular mechanisms. Altogether, our profiling analysis suggests a potential role of RA in neural induction and neuronal differentiation of hAECs (Figure 3).

Our findings suggest that cultured hAECs are able to express stem cell markers, while RA treatment could direct the differentiation of hAECs toward ectodermal lineage. Although the mechanisms of neuronal cell fate determination are not fully elucidated yet, it is apparent that cell-intrinsic programs, including epigenetic regulations as well as TFs and extracellular cues, are involved in this fate specification of stem cells (Schuldiner et al., 2001). Neuronal differentiation is facilitated through promoting proneural genes of neurogenesis and inhibitory mechanisms that maintain self-renewal and proliferation. Remodeling of chromatin structure through histone modification is an important mechanism of modulating gene expression (Murao et al., 2016). We found that RA treatment could regulate histone modification by simultaneously up and downregulating several histone methyltransferases and histone demethylases. Both the activation and repression of histone methylation marks are necessary for the rapid activation of genes during differentiation. Among the histone methyltransferases that were differentially expressed in the treatment hAECs, Eed is the most important. Eed is an active component of PRC2 and was highly expressed in the treated cells. The epigenetic PRC1 promotes proliferation and self-renewal of NSCs, whereas PRC2-mediated gene silencing enhances NSC activation and neurogenesis through histone methylation (van der Vlag and Otte, 1999). Eed suppresses the expression of Gata6 and thereby increases p21 protein level. Gata6 is essential for primitive gut and heart development. On the other hand, increased p21 leads to inhibition of NSC proliferation and self-renewal, and activation of neurogenesis (Sun et al., 2018). Thus, RA may regulate neuroectodermal lineage progression via epigenetic polycomb protein Eed. PRCs together with histone acetylation also affect layer formation of the cortex. Inhibition of HDACs activity decreases the production of deep layer neurons and increases upper layer neurons, and promotes oligodendrocyte production (Namihira et al., 2008). RA treatment downregulated the expressions of *HDAC1* and *HDAC4*. Furthermore, we found upregulation of *BCOR* in the RA-treated hAECS. BCoR can function as a corepressor when tethered to DNA and, when overexpressed, can potentiate BCL-6 repression (Huynh et al., 2000). Specific class I and II HDACs interact with BCoR, suggesting that BCoR may functionally link these two classes of HDACs.

Neuronal differentiation is also regulated by many neural specific bHLH family transcriptional activators and repressors, and the balance of activity between these factors is important for the differentiation process. Helt is a member of bHLH-O transcriptional repressors and contains the characteristic Orange domains. Helt is mainly expressed in the neural progenitors and is down-regulated before neuronal differentiation (Nakatani et al., 2004). Tcf12 (also known as Heb) is a member of the E-box protein family, a subfamily of bHLH protein family members. It plays a critical role during embryonic and adult neurogenesis (Uittenbogaard and Chiaramello, 2002; Mesman and Smidt, 2017). Tcf12 is involved in the control of proliferating stem cells and progenitor cells and early cell fate determination depending on the timing and location of *TCF12* expression. In this study, we found upregulation of *TCF12* expression and downregulation of *HELT* on the 7th day of RA treatment of hAECs.

Epithelial-mesenchymal transition is a common phenomenon during embryonic development. However, EMT during neural induction is not well characterized yet. GO analysis of DEGs revealed highly enriched molecular functions of extracellular matrix component, collagen trimers as well as enriched cellular components of the plasma membrane, cell surface, protein binding, and proteinaceous extracellular matrix (Wang et al., 2012). Together, our GSEA and gene annotation analysis suggests a potential role of RA in EMT and promoting neural induction (Figure 2B and Table 1).

Signaling pathway analysis allows us to understand the molecular basis of biological events that take place during stem cell differentiation. We found that several extracellular pathways were regulated on the 7th day of RA treatment. RA treatment downregulated the expression of several genes related to the canonical WNT pathway, and BMP/TGF-β pathway, whereas upregulated non-canonical effector *NLK*. It has been reported that inhibition of the canonical WNT pathway promotes ESCs differentiation toward ectodermal fate, and activation of the non-canonical WNT pathway enhances neural fate by limiting epidermal choice (Huang et al., 2016). We also found the decreased activity of BMP/TGF-β signaling indicating neural differentiation took place (Doe, 2008). However, most SMAD genes were not changed probably because of their association with EMT during neural induction.

Furthermore, we found that RA treatment could significantly inhibit the Notch pathway. Notch signal pathway has multiple critical roles in the regulation of NSC differentiation and is considered as a molecular switch between neurogenic to gliogenic differentiation of NSCs. Previous evidence suggests that Notch inhibition could promote neuronal commitment and neurogenesis, decreased progenitor pool, and inhibit glial fate differentiation (Zhou et al., 2010; Imayoshi and Kageyama, 2011).

Currently, we are using several semi-biological and synthetic stimulants in neuronal differentiation medium, like neurobasal medium, neural cell supplement N2, non-essential amino acid, BDNF, glial cell line-derived neurotrophic factors, cAMP, ascorbic acid, and laminin. We also use inhibitors of GSK3, TGF-β, and AMPK for neural induction medium. Continuous replacement of this medium makes the whole procedure highly expensive. Therefore, RA would have potential as a cost-effective, easily available alternative stimulant of neuronal differentiation.

Additionally, previous studies showed that transplantation of hAECs into the brain of mice with Parkinson’s disease could significantly increase both the duration of rotation and the number of spontaneous movements (Kong et al., 2008). This suggests that the transplanted hAECs were able to promote endogenous neurogenesis in the SVZ and enhance preservation of the nigrostriatal system. We found that RA treatment could significantly enrich several neurological and psychological diseases, suggesting regulation of genes related to brain diseases. Therefore, RA-induced hAECs may have potential as a new therapeutic approach for several neurodegenerative and psychiatric disorders, including Alzheimer’s disease, Parkinson’s disease, spinal cord injuries, and Huntington’s disease. Furthermore, the differentiation potential of hAECs toward a specific direction using a natural compound unveils the great potential of hAECs as a novel tool for drug screening. However, further in-depth investigations on time and dose-dependent effects of RA treatment on morphology, physiology, and gene expression pattern of hAECs are warranted to confirm its neuronal differentiation potential.

## Data Availability

All data generated or analyzed during this study are included in this published article and its Supplementary Files. Microarray data are deposited in the Gene Expression Omnibus (GEO) under Accession Number: GSE133277 (https://www.ncbi.nlm.nih.gov/geo/info/linking.html).

## Author Contributions

FF investigated the study, responsible for data curation, carried out the formal analysis, visualized the data, and wrote the original draft of the manuscript. KS investigated the study, and carried out the methodology and data validation. YU investigated the study and carried out the formal analysis. NO and HI conceptualized the study, involved in funding acquisition, and carried out the project administration. NO, Y-WZ, and HI were responsible for the resources, supervised the study, and wrote, reviewed, and edited the manuscript. All authors made the substantial contributions to the manuscript and approved its final version.

## Conflict of Interest Statement

The authors declare that the research was conducted in the absence of any commercial or financial relationships that could be construed as a potential conflict of interest.

## Footnotes

1 http://www.affymetrix.com

2 http://software.broadinstitute.org/gsea/index.jsp

## Abbreviations

bHLH: basic helix-loop-helix
cRNA: complementary RNA
DEGs: differentially expressed genes
EMT: epithelial-mesenchymal transition
ESCs: embryonic stem cells
GAD: genetic association database
GO: gene ontology
hAECs: human amnion epithelial cells
HDAC: histone deacetylase
iPSCs: induced PSCs
MSCs: mesenchymal stem cells
PRC: polycomb repressive complex
PSCs: pluripotent stem cells
RA: rosmarinic acid.
