# Supplementary material for: Exploring the Potential Role of Rosmarinic Acid in Neuronal Differentiation of Human Amnion Epithelial Cells by Microarray Gene Expression Profiling
Source: Front Neurosci. 2019 Jul 24;13:779. doi: 10.3389/fnins.2019.00779 (PMC6667736; doi:10.3389/fnins.2019.00779)
Supplement: Supplementary file 1 [file Data_Sheet_1.zip › Final Supplementary materials/Supplementary Figure 2.pdf]

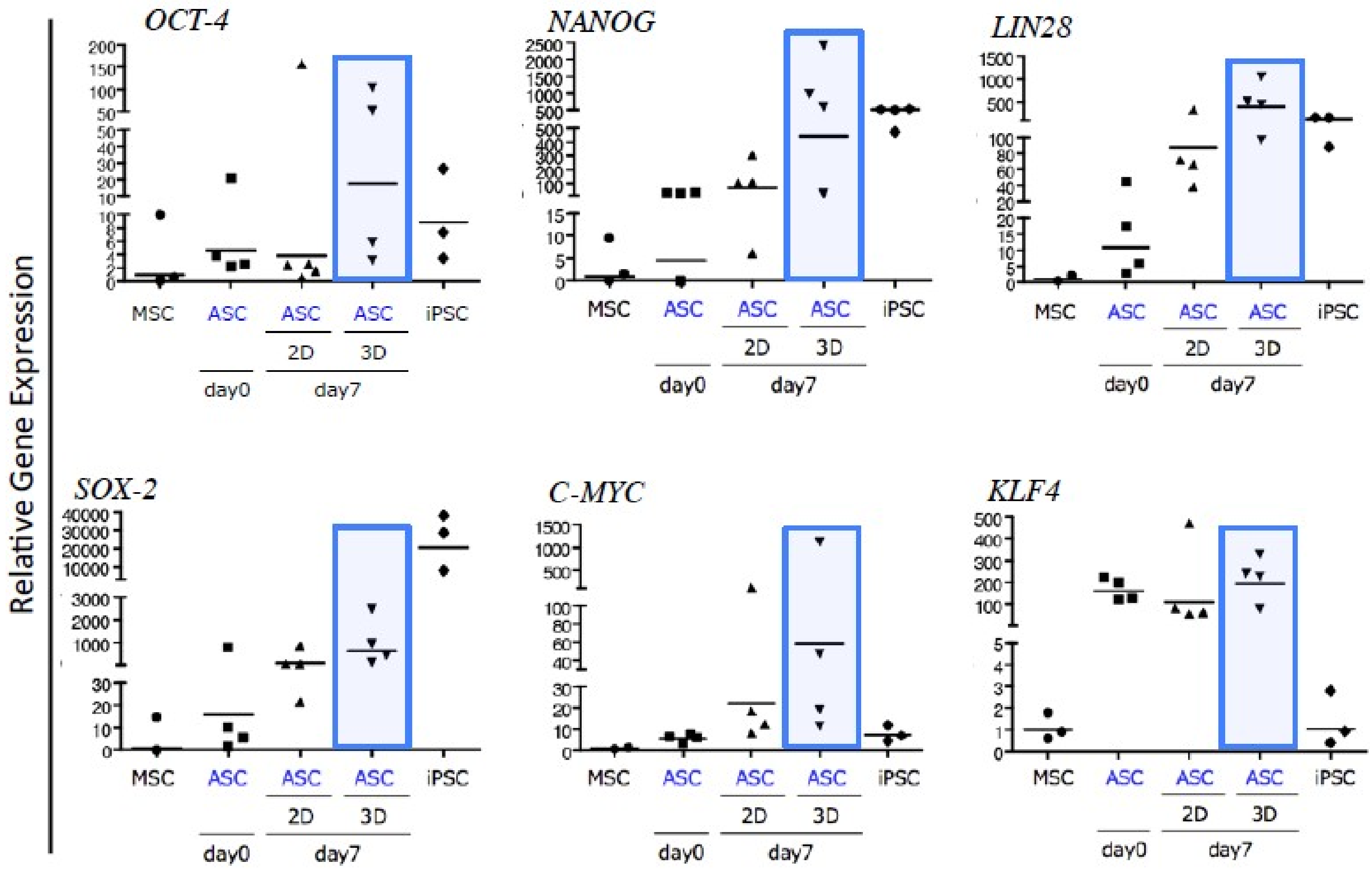

Supplementary Figure 2: AEC spheroids (3D culture) highly expressed the stem-ness related gene markers compared to the 2D counterpart [MSC: mesenchymal stem cell; ASC: amniotic epithelial (stem) cell, iPSC: induced pluripotent stem cell]
