# Supplementary material for: Exploring the Potential Role of Rosmarinic Acid in Neuronal Differentiation of Human Amnion Epithelial Cells by Microarray Gene Expression Profiling
Source: Front Neurosci. 2019 Jul 24;13:779. doi: 10.3389/fnins.2019.00779 (PMC6667736; doi:10.3389/fnins.2019.00779)
Supplement: Supplementary file 1 [file Data_Sheet_1.zip › Final Supplementary materials/Supplementary Figure 3.pdf]

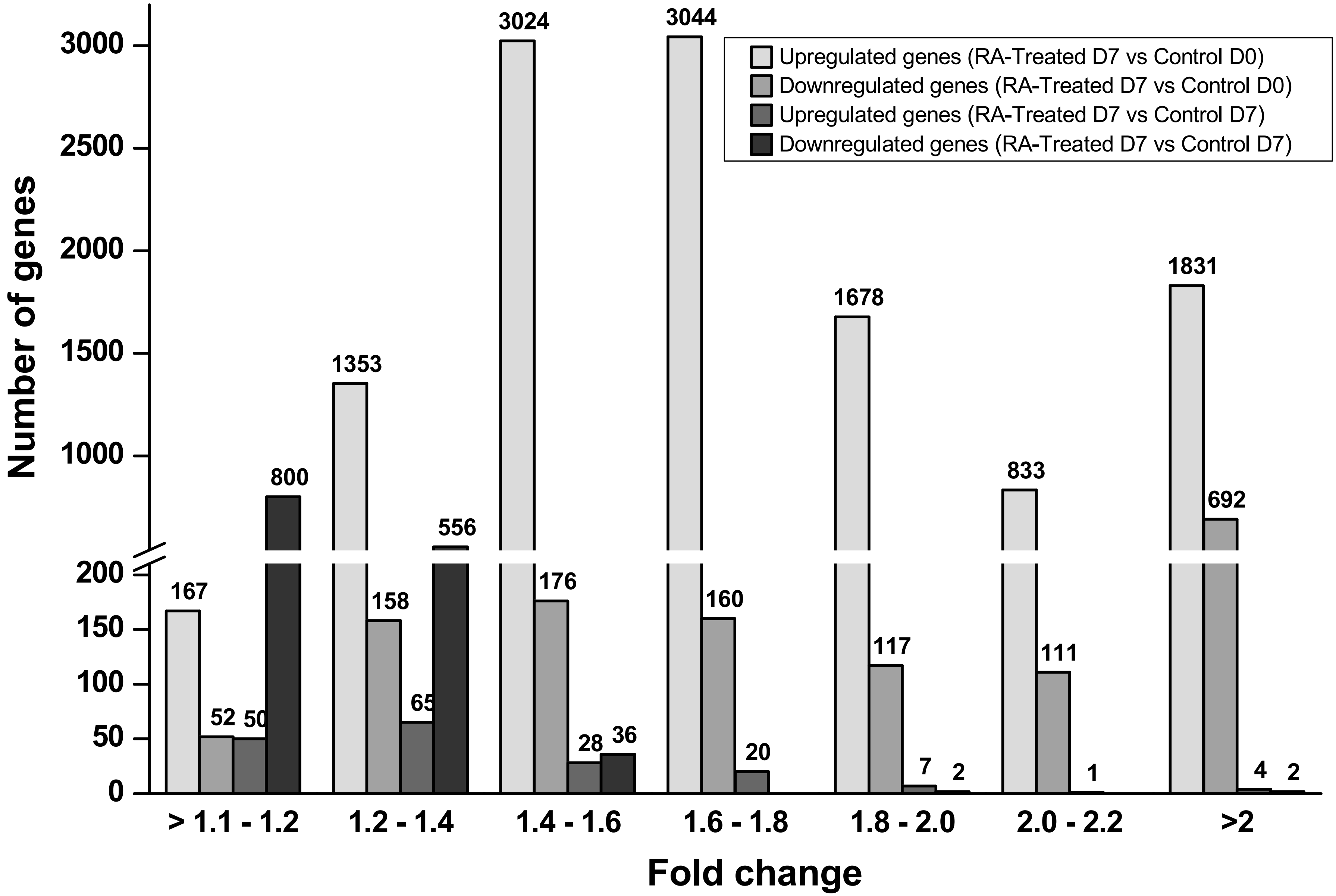

Supplementary Figure 3: Distribution of fold changes in mRNA expressions between RA-treated and untreated control hAECs
