# Supplementary material for: Exploring the Potential Role of Rosmarinic Acid in Neuronal Differentiation of Human Amnion Epithelial Cells by Microarray Gene Expression Profiling
Source: Front Neurosci. 2019 Jul 24;13:779. doi: 10.3389/fnins.2019.00779 (PMC6667736; doi:10.3389/fnins.2019.00779)
Supplement: Supplementary file 1 [file Data_Sheet_1.zip › Final Supplementary materials/Supplementary Figure 4.pdf]

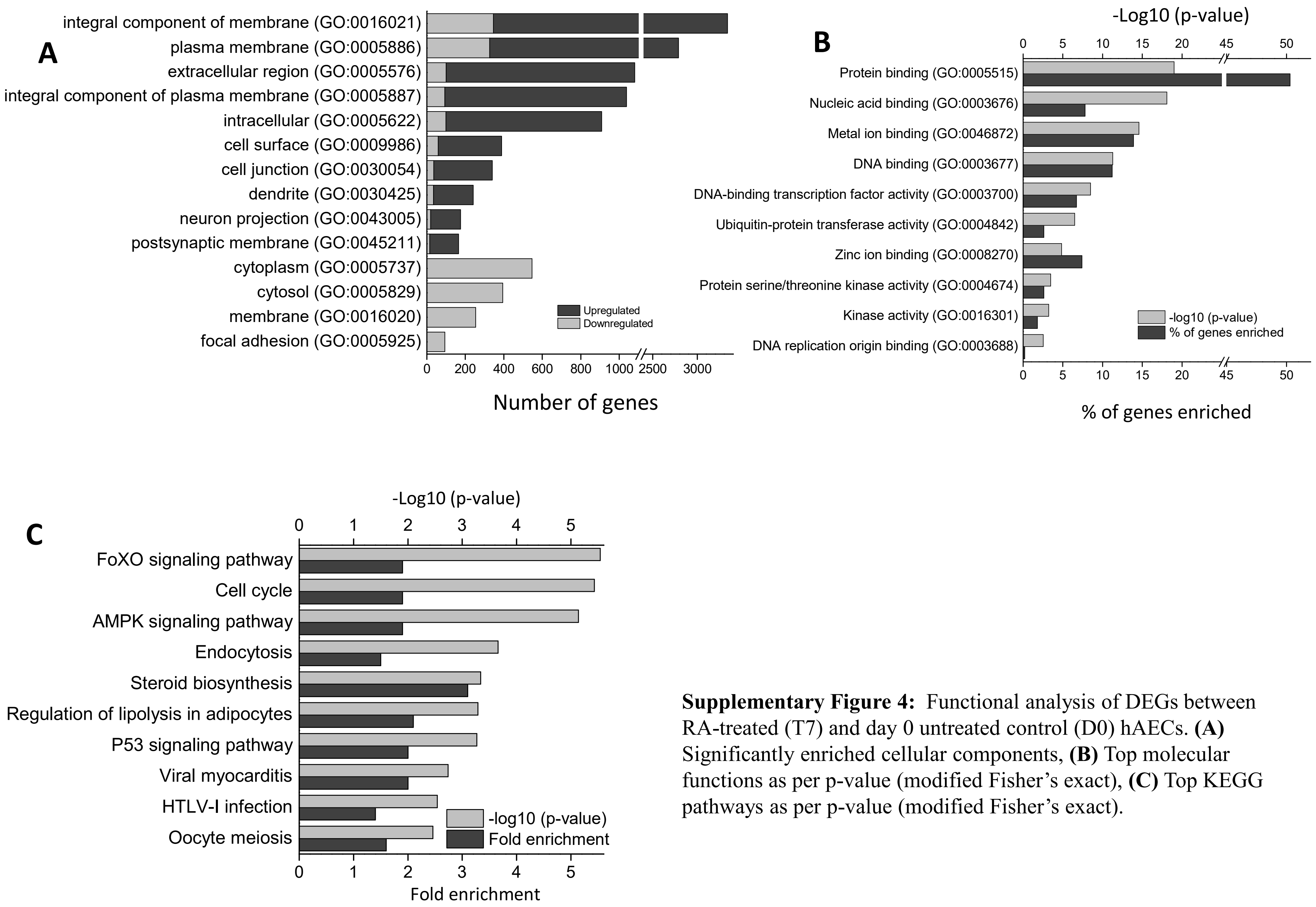

Supplementary Figure 4: Functional analysis of DEGs between RA-treated (T7) and day 0 untreated control (D0) hAECs. **(A)** Significantly enriched cellular components, **(B)** Top molecular functions as per p-value (modified Fisher's exact), **(C)** Top KEGG pathways as per p-value (modified Fisher's exact).
