# Supplementary material for: Exploring the Potential Role of Rosmarinic Acid in Neuronal Differentiation of Human Amnion Epithelial Cells by Microarray Gene Expression Profiling
Source: Front Neurosci. 2019 Jul 24;13:779. doi: 10.3389/fnins.2019.00779 (PMC6667736; doi:10.3389/fnins.2019.00779)
Supplement: Supplementary file 1 [file Data_Sheet_1.zip › Final Supplementary materials/Supplementary Table 1.pdf]

Supplementary table 1: List of important DEGs

Gene Symbol	Description	Fold Change	p-value	Fold Change	p-value
	**List of top ten upregulated genes (T7 vs D0)**	**T7 vs D0**		**T7 vs D7**	
ARRDC4	arrestin domain containing 4	43.0	0.006	--	--
GPNMB	glycoprotein (transmembrane) nmb	32.21	0.00008	--	--
CYP1A1	cytochromeP450, family1, subfamilyA, polypeptide 1	26.63	0.022	--	--
TXNIP	thioredoxin interacting protein	25.58	0.0004	--	--
CSTA	cystatin A (stefin A)	21.76	0.002	--	--
TGFBR3	transforming growth factor, beta receptor III	20.91	0.047	--	--
TTC32	tetratricopeptide repeat domain 32	19.54	0.007	--	--
ID2	inhibitor of DNA binding 2, dominant negative helix-loop-helix protein	19.36	0.045	--	--
KRTDAP	keratinocyte differentiation-associated protein	17.63	0.001	--	--
ZNF816	zinc finger protein 816	16.42	0.014	--	--
	**List of top ten downregulated genes (T7 vs D0)**				
SERPINE2	serpin peptidase inhibitor, clade E, member 2	-130.41	0.000003	--	--
AXL	AXL receptor tyrosine kinase	-108.92	0.00007	--	--
C4orf26	chromosome 4 open reading frame 26	-76.31	0.0001	--	--
PAPPA	pregnancy-associated plasma protein A	-75.62	0.00004	--	--
CYR61	cysteine-rich, angiogenic inducer, 61	-46.31	0.0002	--	--
TYMS	thymidylate synthetase	-44.70	0.0014	--	--
KISS1	KiSS-1 metastasis-suppressor	-40.42	0.00005	--	--
LTBP2	latent transforming growth factor beta binding protein 2	-40.17	0.0007	--	--
INHBA	inhibin, beta A	-36.10	0.00013	-1.29	0.018
SERPINE1	serpin peptidase inhibitor, clade E, member 1	-32.45	0.004	--	--
	**List of top ten upregulated genes (T7 vs D7)**				
METTL3	methyltransferase like 3	1.75	0.008	2.32	0.049
BTBD1	BTB (POZ) domain containing 1	1.60	0.011	2.29	0.007
AP3M1	adaptor-related protein complex 3, mu 1 subunit	1.84	0.002	2.28	0.019
RAB6A	RAB6A, member RAS oncogene family	1.46	0.026	2.28	0.038
CREG1	cellular repressor of E1A-stimulated genes 1	2.05	0.003	2.05	0.037
RPE	cRPE; ribulose-5-phosphate-3-epimerase	1.80	0.009	1.96	0.025
KLHL42	kelch-like family member 42	--	--	1.95	0.016
VAMP3	vesicle-associated membrane protein 3	1.18	0.023	1.92	0.018
GYG1	glycogenin 1	1.52	0.010	1.91	0.023
WDR1	WD repeat domain 1	-4.05	0.005	1.89	0.024
	**List of top ten downregulated genes (T7 vs D7)**				
HIST2H2AC	histone cluster 2, H2AC	2.26	0.015	-2.6	0.044
DDX6	DEAD (Asp-Glu-Ala-Asp) box helicase 6	2.13	0.022	-2.42	0.011
RBAK	RB-associated KRAB zinc finger	2.83	0.009	-1.94	0.044
PRDM2	PR domain containing 2, with ZNF domain	2.98	0.004	-1.83	0.011
AKT2	v-akt murine thymoma viral oncogene homolog 2	-2.52	0.011	-1.55	0.005
ZKSCAN1	zinc finger with KRAB and SCAN domains 1	2.22	0.004	-1.53	0.043
GPR31	G protein-coupled receptor 31	--	--	-1.5	0.007
MMP28	matrix metallopeptidase 28	1.37	0.00001	-1.49	0.017

TMPRSS4	transmembrane protease, serine 4	1.74	0.004	-1.49	0.007
FLJ33360	FLJ33360 protein	1.91	0.007	-1.47	0.045
	**Endoderm Markers genes**				
GATA1	GATA binding protein 1	1.49	0.014	-1.11	0.011
HNF4A	hepatocyte nuclear factor 4, alpha	1.59	0.006	-1.11	0.046
NKX3-1	NK3 homeobox 1	1.60	0.008	-1.21	0.009
SOX17	SRY (sex determining region Y)-box 17	1.45	0.033	-1.25	0.031
	**Mesoderm marker genes**				
FOXF1	forkhead box F1	1.57	0.011	-1.11	0.013
MESP1	mesoderm posterior 1 homolog	1.42	0.008	-1.12	0.026
DLL3	delta-like 3	1.43	0.006	-1.13	0.005
MESDC1	mesoderm development candidate 1	1.40	0.0001	-1.14	0.023
MEOX1	mesenchyme homeobox 1	1.51	0.004	-1.23	0.029
	**Ectoderm-related genes**				
OTX2	orthodenticle homeobox 2	1.81	0.002	--	--
SOX1	SRY (sex determining region Y)-box 1	1.57	0.014	--	--
PAX6	paired box 6	1.72	0.007	--	--
EED	embryonic ectoderm development	1.64	0.003	1.73	0.040
DPPA4	developmental pluripotency associated 4	1.68	0.039	-1.32	0.015
	**Transcription Factors**				
TCF12	transcription factor 12	1.93	0.0002	1.63	0.028
HNRNPD	heterogeneous nuclear ribonucleoprotein D	2.80	0.008	1.28	0.047
NLK	nemo-like kinase	1.93	0.004	1.17	0.034
SMAD5	SMAD family member 5	2.74	0.009	1.15	0.044
HELT	helt bHLH transcription factor	1.49	0.043	-1.23	0.027
ASCL1	achaete-scute complex homolog 1	1.67	0.0008	-1.24	0.019
TCF7L1	transcription factor 7-like 1	1.40	0.033	-1.13	0.032
FOXE3	forkhead box E3	--	--	-1.2	0.044
EHF	ets homologous factor	1.69	0.004	-1.11	0.035
ZBTB17	zinc finger and BTB domain containing 17	1.49	0.009	-1.23	0.018
MAML1	mastermind-like 1	1.55	0.012	-1.21	0.022
HOXC13	homeobox C13			-1.3	0.031
PAX2	paired box 2	1.63	0.014	-1.15	0.019
SP8	Sp8 transcription factor	1.50	0.005	-1.14	0.025
EGR1	early growth response 1	1.56	0.003	-1.26	0.009
HEYL	hairy/enhancer-of-split related with YRPW motif-like	1.82	0.004	-1.44	0.025
	**Epigenetic Modifiers**				
PRMT6	protein arginine methyltransferase 6	-1.58	0.008	1.17	0.033
KMT2C	lysine (K)-specific methyltransferase 2C	1.51	0.018	1.31	0.038
PRDM2	PR domain containing 2, with ZNF domain	2.98	0.004	-1.83	0.011
SETD3	SET domain containing 3	2.41	0.014	-1.24	0.004
KDM2B	lysine (K)-specific demethylase 2B	-1.36	0.002	1.12	0.032
KDM5A	lysine (K)-specific demethylase 5A	2.21	0.004	-1.2	0.025
HDAC1	histone deacetylase 1	1.50	0.010	-1.15	0.025
HDAC4	histone deacetylase 4	1.41	0.003	-1.11	0.023
SIRT6	sirtuin 6	1.43	0.009	-1.23	0.039

	**EMT-related genes**				
CDH6	cadherin 6, type 2, K-cadherin (fetal kidney)	--	--	1.47	0.046
CDH16	cadherin 16, KSP-cadherin	1.40	0.027	-1.32	0.004
CDHR5	cadherin-related family member 5	1.93	0.0007	-1.21	0.044
COL11A1	collagen, type XI, alpha 1	1.76	0.0007	-1.14	0.010
COL13A1	collagen, type XIII, alpha 1	2.41	0.015	-1.18	0.035
COL14A1	collagen, type XIV, alpha 1	1.68	0.011	-1.14	0.019
COL4A1	collagen, type IV, alpha 1	-4.56	0.004	-1.13	0.021
COL4A2	collagen, type IV, alpha 2	-3.61	0.00008	-1.19	0.030
COL9A1	collagen, type IX, alpha 1	1.49	0.004	-1.14	0.049
CLDN18	claudin 18	2.10	0.013	-1.13	0.049
EPB41L5	erythrocyte membrane protein band 4.1 like 5	4.31	0.003	-1.22	0.034
KAZN	kazrin, periplakin interacting protein	1.52	0.022	-1.23	0.012
CD74	CD74 molecule	-2.28	0.001	-1.12	0.008
DCSTAMP	dendrocyte expressed seven transmembrane protein	--	--	-1.12	0.011
CSPG4	chondroitin sulfate proteoglycan 4			-1.26	0.021
ARHGAP24	Rho GTPase activating protein 24	1.78	0.012	-1.17	0.009
FYCO1	FYVE and coiled-coil domain containing 1	4.21	0.009	-1.25	0.048
SPIRE 1	spire homolog 1			-1.11	0.018
	**WNT pathway-related genes**				
BTRC	beta-transducin repeat containing E3 ubiquitin protein ligase	3.07	0.003	-1.25	0.043
CCNY	cyclin Y	2.05	0.009	-1.41	0.049
FZD5	frizzled family receptor 5	1.25	0.008	-1.22	0.010
RSPO1	R-spondin 1	1.65	0.0007	-1.14	0.044
WNT5B	wingless-type MMTV integration site family, member 5B	1.41	0.009	-1.26	0.023
WISP3	WNT1 inducible signaling pathway protein 3	1.48	0.0003	-1.11	0.044
	**BMP/TGF-β signaling pathway related genes**				
ACVRL1	activin A receptor type II-like 1	1.28	0.019	-1.23	0.047
ASPN	asporin	1.68	0.009	-1.39	0.015
CHRD	chordin	1.64	0.009	-1.13	0.047
SLC39A5	solute carrier family 39 (metal ion transporter), member 5	1.43	0.010	-1.23	0.030
TGF-β2	transforming growth factor, beta 2	-2.21	0.008	-1.13	0.028
BMP8A	bone morphogenetic protein 8a	1.61	0.011	-1.25	0.017
BMP8B	bone morphogenetic protein 8b	1.50	0.012	-1.12	0.030
BMPER	BMP binding endothelial regulator	--	--	-1.2	0.009
	**Notch Signaling pathway**				
NOTCH2	notch 2	1.53	0.049	-1.2	0.041
DLL1	delta-like 1	1.85	0.002	-1.17	0.041
DLL3	delta-like 3	1.43	0.005	-1.13	0.005
DTX1	deltex homolog 1	1.55	0.026	-1.25	0.018
HEYL	hairy/enhancer-of-split related with YRPW motif-like	1.82	0.004	-1.44	0.025
SBNO2	strawberry notch homolog 2	1.61	0.012	-1.13	0.004

PKMYT1	protein kinase, membrane associated tyrosine/threonine 1	1.59	0.004	-1.32	0.019
METTL3	methyltransferase like 3	1.75	0.008	2.32	0.049
